# Development of invasive non-typhoidal *Salmonella* conjugate vaccines and their evaluation in a trivalent formulation with typhoid conjugate vaccine

**DOI:** 10.1016/j.vaccine.2025.126913

**Published:** 2025-04-11

**Authors:** So Jung An, Jae Seung Yang, Myung Hwa Chae, Joo Sung Woo, Ye Eun Kang, Ravi Ganapathy, Ruchir Kumar Pansuriya, Jung Ah Choi, Yeon Kyung Yoon, Eugene Lee, Seul Bee Lee, Gaurav Pandey, Ji Won Lee, Ji Soo Lee, So Hee Bae, Soh-Won Kweon, Soo Ji Kim, Seung Han Seon, Jerome H. Kim, Manki Song

**Affiliations:** International Vaccine Institute, SNU Research Park, 1 Gwanak-ro, Gwanak-gu, Seoul, Republic of Korea

**Keywords:** iNTS, O-specific polysaccharide, Typhoid fever, Vi capsular polysaccharide, Conjugate vaccine, Trivalent iNTS/typhoid vaccine

## Abstract

Invasive nontyphoidal *Salmonella* (iNTS) infections, primarily caused by *Salmonella enterica* serovars Typhimurium (*S.* Typhimurium) and Enteritidis (*S.* Enteritidis), represent a significant public health concern, particularly in sub-Saharan Africa, where multidrug-resistant (MDR) strains are increasingly prevalent. Despite the substantial disease burden, no vaccines are currently licensed for iNTS. This study aimed to develop an iNTS conjugate vaccine by conjugating O-specific polysaccharide (OSP) antigens to carrier proteins using chemical conjugation, a proven method known for its efficiency and scalability in licensed glycoconjugate vaccines. Various carrier proteins and chemical conjugation processes were evaluated to optimize the iNTS OSP conjugate vaccine candidates. Through this optimization, diphtheria toxoid (DT) was identified as the carrier protein that significantly enhances the anti-OSP immunogenicity of the iNTS conjugates. Key properties, such as the molecular weight and OSP:DT ratio in the iNTS OSP conjugate were found to be controllable by adjusting the ratios of CDAP conjugate reagent and DT to iNTS OSP. Optimal conjugation process parameters were identified by evaluating the relationship between these property and immunogenicity through tests in mice. The optimized iNTS conjugates for *S.* Typhimurium and *S.* Enteritidis were further developed into a bivalent formulation. This formulation was selected based on a dose-dependent immunogenicity study and included alum as an adjuvant to enhance immune response. Ultimately, a trivalent drug product formulation was developed by combining the bivalent iNTS conjugate vaccine with a typhoid conjugate vaccine.

Our findings demonstrated that the iNTS OSP-DT conjugates, at the optimal conjugation ratios, induced robust immune responses with high anti-OSP IgG titers for both iNTS serovars, comparable to or exceeding those of other formulations. The inclusion of alum further enhanced immunogenicity across all formulations. Notably, the trivalent vaccine formulation showed promising results, maintaining robust immunogenic responses against all iNTS OSP antigens and the Vi polysaccharide antigen of *Salmonella* Typhi, without compromising the immunogenicity of any individual antigens.

This study suggests that a bivalent iNTS vaccine combined with a typhoid conjugate vaccine could provide broad protection against both iNTS infections and typhoid fever, addressing a critical unmet need in regions with limited resources.

## 1 Introduction

Invasive nontyphoidal *Salmonella* infections represent a significant global health threat, particularly in sub-Saharan Africa and other low-resource settings [1,2]. Unlike typical non-invasive *Salmonella* infections, which primarily cause gastroenteritis, iNTS can lead to severe systemic infections, such as bacteremia and meningitis, with symptoms ranging from fever and diarrhea to severe systemic illness. The primary pathogens responsible for iNTS are *Salmonella* Typhimurium and *Salmonella* Enteritidis, which can cause severe systemic infections, particularly in vulnerable populations [[3], [4], [5]]. Recent estimates indicate that iNTS infections cause approximately 535,000 cases and 77,500 annual deaths worldwide, with the highest burden observed in sub-Saharan Africa. In these regions, children under five years old and individuals with compromised immune systems, such as those infected with HIV, are mainly affected [6,7]. The increasing prevalence of multidrug-resistant (MDR) strains is a growing concern for the management of iNTS infections. MDR iNTS strains complicate the treatment regimens and increase the risk of severe outcomes by limiting the efficacy of commonly used antibiotics. This situation underscores the urgent need for effective vaccines to prevent these infections and mitigate the impact of antimicrobial resistance [8,9].

Despite the significant health burden imposed by iNTS, no licensed vaccines are currently available. This gap in vaccine availability contrasts sharply with the existence of vaccines for *Salmonella* Typhi, such as the Vi polysaccharide and Vi conjugate vaccines, which have proven to be effective in preventing typhoid fever [10] . Recent studies have focused on developing vaccines targeting *S.* Typhimurium (ST) and *S.* Enteritidis (SE), which are responsible for most iNTS cases. O-specific polysaccharide (OSP), which is isolated from lipopolysaccharide (LPS) and excludes lipid A on the *Salmonella* surface, has shown promise as a vaccine antigen against iNTS, especially in conjugate vaccines [11]. Previous preclinical studies have demonstrated that OSP conjugates induce protective immunity against lethal challenges, and that anti-OSP IgG and IgM are responsible for protection against passive immunization [[12], [13], [14], [15]]. Notably, a conjugate vaccine based on OSP chemically linked to the flagellin protein (FliC) from homologous serovars of *Salmonella* has shown immunogenicity and protective efficacy in preclinical models and has gone through a Phase 1 clinical trial [16]. Additionally, a preclinical study on a Generalized Modules for Membrane Antigens (GMMA)-based iNTS glycoconjugate has shown promising immune responses, and the glycoconjugate is currently undergoing phase 1 clinical trials [17]. Another promising approach involved a bivalent glycoconjugate vaccine incorporating OSP using a Multiple Antigen-Presenting System (MAPS), which has demonstrated robust immune responses in animal models  [18].

To develop an iNTS bivalent conjugate vaccine, our primary goal was to produce a highly effective and affordable vaccine suitable for use in low- and middle-income countries (LMICs). To achieve this, we used 1-cyano-4-dimethylaminopyridinium tetrafluoroborate (CDAP) conjugation chemistry, a method commonly used for commercial vaccines, along with existing carrier proteins. This platform is cost-effective and facilitates the transfer of conjugate vaccines to LMICs. CDAP has been used in several commercial conjugate vaccines, including those against *Haemophilus influenzae* type b (Hib) and pneumococcal infections. These vaccines have demonstrated the robustness and reliability of CDAP chemistry in vaccine production, offering advantages in producing high-yield, low-cost vaccines, making them an ideal choice for large-scale vaccine manufacturing, especially in resource-limited settings. The use of diphtheria toxoid (DT) or tetanus toxoid (TT) as carrier proteins reduces the overall cost of vaccines.

This study aimed to identify optimal elements for producing iNTS OSP conjugate vaccines using CDAP conjugation chemistry. We evaluated various conjugation process factors, including carrier protein selection, conjugation ratios, and the physicochemical properties of the resulting conjugates. The immunogenicity of the optimal iNTS SE OSP and ST OSP conjugates was evaluated for both monovalent and bivalent iNTS conjugate vaccine formulations in mice. Furthermore, we demonstrated that trivalent vaccine formulation, including the Vi-DT typhoid conjugate as a drug product (DP) multi-dose presentation for LMICs, could be effectively developed using the optimized DP composition.

## 2 Materials and methods

### 2.1 Materials

The following chemicals were used in this study: Yeast extract UF (BD, NJ, USA), Casamino acid (BD), Na_2_HPO_4_.7H_2_O (Sigma-Aldrich, MO, USA), NaH_2_PO_4_.H_2_O (Sigma-Aldrich), MgSO_4_.7H_2_O (Sigma-Aldrich), CDAP (Sigma-Aldrich,), 1-ethyl-3-(3-dimethylaminopropyl) carbodiimide hydrochloride (EDC) (Sigma-Aldrich), adipic acid dihydrazide, acetonitrile, sodium tetraborate decahydrate sodium borate, sodium hydroxide triethylamine (TEA), sodium hydroxide, glycine, 2-(N-Morpholino) ethane sulfonic acid (“MES”) buffer, phosphate-buffered saline (PBS), 2-phenoxyethanol, succinic acid, aluminum phosphate (Adju-Phos®) (Invivogen, MA, USA), abequose (Toronto research chemicals, Ontario, Canada), tyvelose (Santa Cruz Biotechnology), and glucose, galactose, rhamnose, mannose, and *N*-acetyl glucosamine (all from Sigma-Aldrich).

### 2.2 Carrier proteins

DT was provided by SK bioscience (Seongnam-si, Gyeonggi-do, Republic of Korea), and TT was purchased from PT Bio Farma (Bandung, Indonesia). The Vi-DT conjugate was supplied by SK bioscience, with all quality attributes meeting the acceptance criteria, as confirmed by the Certificate of Analysis.

### 2.3 O-specific polysaccharides

O-specific polysaccharides were produced and purified from clinical isolate GNB00128 from Guinea Bissau for *Salmonella enterica* Enteritidis (SE) and isolate 114,575 from Ghana for *Salmonella enterica* Typhimurium (ST) from the International Vaccine Institute biorepository in our previous study [19]. ST and SE OSPs were produced by cultivating the respective strains in a 5 L Biostat B Sartorius fermenter using a modified media composition, as previously described [20] with a modified feeding strategy. The basal media composition was 3 g/L glucose, 10 g/L yeast extract UF, 5 g/L casamino acid, 18.8 g/L Na_2_HPO_4_.7H_2_O, 4.1 g/L NaH_2_PO_4_.H_2_O, and 1.75 g/L MgSO4.7H_2_O. The feed medium comprised 100 g/L yeast extract, 100 g/L casamino acids, and 300 g/L glucose. All media components were sterilized by 0.2 μm filtration (Sartopore 2300). The fermentation was performed at a temperature of 35 ± 1 °C, pH 7.2 ± 0.1 (maintained using 3 N NaOH and feed solution), DO maintained at 35 % in cascade with an agitator, air, and oxygen. Feeding was continued until the OD600nm reached 80 for ST and 120 for SE. The culture was inactivated by formaldehyde by incubating for 14 ± 2 h at 25 °C and 200 RPM.

The purification of OSPs was adopted and modified from previous studies by Kothari et al. and Micoli et al. [11,20]. The inactivated culture was clarified using 0.2 μm tangential flow filtration (TFF), with the retentate diafiltered against a 20× volume of saline solution. The clarified cells were subjected to hydrolysis using 1 % acetic acid, at 100 °C for one hour, and the acid-hydrolyzed material was clarified using a depth filter. Impurities were precipitated using 0.2 % *w*/*v* sodium deoxycholate at a lower pH. The precipitate containing the impurities was captured in a 0.45 μm capsule filter, and the clear supernatant was subjected to TFF using 30-kDa cut-off membrane-cassette and diafiltered 40 times against 50 mM Tris-HCl, pH 5.75 buffer. The diafiltered solution was subjected to cation-exchange chromatography using an Eshmuno CPX resin (Merck). Briefly, a 25 mL CEX resin column was equilibrated with 3CV of 50 mM Tris-HCl, pH 5.75 buffer, the diafiltered OSP was injected at a flow rate of 5 mL/min, and the unbound ST/SE OSP was collected as flow-through (FT). Impurities were eluted with 3CVs each with 20 % and 100 % buffer having 50 mM Tris-acetate/1 M NaCl. Lastly, TFF using a 30-kDa cut-off membrane cassette was performed for the FT, including 20 volumes of diafiltration against purified water. The resultant purified OSP was sterile-filtered using a 0.2 μM sterile filter (Sartopore 2300) and stored at −20 °C until use.

### 2.4 Preparation of iNTS OSP conjugates

The conjugation chemistries used for *S.* Typhimurium and *S.* Enteritidis OSP-DT or OSP-TT evaluation were selected and modified based on methods from previous studies [12] [21]. OSP conjugation was performed using an organic cyanylating reagent, CDAP, to activate OSP in preparation for coupling a carrier protein, DT or TT by two synthetic methods with (indirect) or without hydrazide (-AH) modification (direct), to the OSP (Table 1 and Table 2). Details of conjugate syntheses are given in the supplementary material (Fig. S1 and S2).

Monovalent and bivalent iNTS conjugates were formulated in PBS at pH 7.4, with varying doses based on the test design (Table 3). The trivalent iNTS/Typhoid vaccine formulation for mice, per 0.1 mL dose, included 5 μg of SE OSP, 2.5 μg of ST OSP, and 2.5 μg of Vi polysaccharide, all conjugated to DT. Additionally, 10 mg/mL 2-phenoxyethanol was added as a preservative for multi-dose presentation in a drug product vial. The buffer system of the trivalent formulation contained 39.9 μg of succinic acid per dose (3.38 mM), 2.5 μg of potassium phosphate, monobasic (0.19 mM), 7.4 μg of sodium phosphate, dibasic (0.52 mM), and 0.75 mg of sodium chloride (129.31 mM) to maintain isotonicity. All formulations with alum included 0.07 mg of aluminum per dose, which served as an adjuvant at a concentration of 0.7 mg/mL.

### 2.5 OSP and conjugate characterization

The purified ST and SE OSPs were characterized for various impurities such as residual protein (Lowery assay), residual nucleic acids (absorbance at 260 nm using spectrophotometry), and endotoxin content (LAL method). OSP was quantified using an anthrone assay, and purity was confirmed by SEC-HPLC using an RI detector [11]. Samples were run on a TSK gel G3000 PWXL column (Tskgel G3000PWXL; 7.8 mm × 30 mm; particle size 7 μm) with TSK gel PWXL guard column (4.0 cm × 6.0 mm; particle size 12 μm) (Tosoh Bioscience, Griesheim. Germany). The mobile phase was 0.1 M NaCl, 0.1 M NaH2PO4, 5 % CH3CN, pH 7.2 at the flow rate of 0.5 mL/min (isocratic method for 30 min). Void and bed volume calibration was performed with k-DNA (k-DNA Molecular Weight Marker III 0.12–21.2 Kbp, Roche) and sodium azide (NaN3), respectively, and dextrans were used as standards.

Conjugates were evaluated for OSP quantification using the anthrone assay, while both identification and quantification of OSP in bivalent and trivalent formulations were performed using high-performance anion-exchange chromatography (HPAEC-PAD) (on a Dionex CarboPac PA1 column (4 × 250 mm) with a Dionex AminoTrap column (4 × 50 mm) (ICS-5000+, Thermo Scientific, MA, USA) as described by Micoli et al. [11]. The amounts of DT and TT were determined using Lowry's assay. The molecular size distribution was assessed by high-performance liquid chromatography-size-exclusive chromatography (HPLC-SEC) using a Tskgel G5000PWXL (7.8 mm × 30 mm, Tosoh Bioscience) with a TSK gel PWXL guard column. The average molecular weight was measured using multi-angle light scattering (MALS) with a HELEOS II detector (Wyatt Technology, CA, USA) using a 12-kDa dextran standard for normalization.

### 2.6 Assessment of immunogenicity

Anti-OSP IgGs against *S.* Typhimurium and *S.* Enteritidis and anti-Vi IgG in mouse serum were measured using an enzyme-linked immunosorbent assay. Briefly, 96-well plates (Nunc, 439,454) were coated with 100 μL of OSP antigen (2 μg/mL) of *S.* Typhimurium or *S.* Enteritidis and Vi of *S.* Typhi in phosphate-buffered saline (PBS) at 4 °C overnight. After washing, non-specific binding was blocked with 5 % skim milk in PBS for 1 h at room temperature. The plates were washed, and 2-fold serially diluted mouse serum was added to the microwells and incubated for 1 h at room temperature. After washing the plates, alkaline phosphatase-conjugated goat anti-mouse IgG (Southern Biotechnology) was added and incubated for 1 h at room temperature. The plates were washed and developed by adding 1 mg/mL 4-nitrophenyl phosphate substrate (Sigma-Aldrich, USA). Optical densities were measured at 405 nm and corrected at 490 nm. Anti-Vi IgG antibody was assessed using ELISA as previously described [22] except for the definition of antibody titer using an internal mouse control. Antibody titers were determined as endpoint titers, which were expressed as the reciprocal log_2_ of the last dilution giving an OD at 405 nm of 0.15 or as arbitrary units per ml (AU/mL) based on each internal standard serum. The limits of quantitation for anti-ST, -SE OSP, and -Vi IgGs were 0.05 AU/mL, 0.02 AU/mL, and 0.01 AU/mL, respectively.

### 2.7 Animal experiments

All experiments were approved by the Institutional Animal Care and Use Committee of the International Vaccine Institute (IACUC approval No. 2018–025, 2020–003). We purchased 4–6-week-old female ICR (CD-1) mice from Koatech (Pyeongtaek-si, Gyeonggi-do, Republic of Korea); mice were acclimatized for one week prior to the experiments. Ten mice per group were immunized with 100 μL of each formulation via intramuscularly three times at 2-week intervals in all animal experiments. Serum samples obtained at 4 weeks after 3rd immunization were assessed for IgGs against ST OSP, SE OSP, and Vi using ELISA. The formulations prepared for each test are listed in Tables 1, 2, and 3. Four weeks after the final immunization, blood was collected from the retro-orbital plexus of anesthetized mice. The serum was separated from the blood by centrifugation at 3000 rpm for 40 min at 4 °C and stored at −80 °C until use.

### 2.8 Statistical analysis

Statistical analyses were conducted using GraphPad Prism 10 software (version 10.4.1). For comparisons involving multiple groups, one-way ANOVA with Tukey's post-hoc test or Kruskal-Wallis analysis with Dunn's post-hoc test was applied, depending on the data distribution. A two-tailed *t*-test was used for comparison between the two groups.

## 3 Results

### 3.1 Conjugation and immunogenicity in mice to select carrier protein of iNTS O-specific polysaccharide conjugates

To identify the optimal carrier protein and conjugation method using CDAP chemistry, ST and SE OSP were conjugated to both modified (hydrazide-derivatized) and unmodified forms of DT and TT. The results showed that hydrazide-derivatized DT (DTAH) and TT (TTAH) produced conjugates with larger molecular weights than the direct conjugation with unmodified carrier proteins. Specifically, conjugates with derivatized TT (TTAH) exhibited the highest average molecular weights for both ST and SE OSP conjugates, with sizes of 4560 kDa and 5871 kDa, respectively. (Table 1). The OSP-to-protein ratio (w/w) was similar for both the modified and unmodified protein conjugates, indicating consistent conjugation efficiency. The OSP yield was consistently greater than 60 % for all conjugates, demonstrating effective conjugation using CDAP chemistry.

**Fig. 1:**
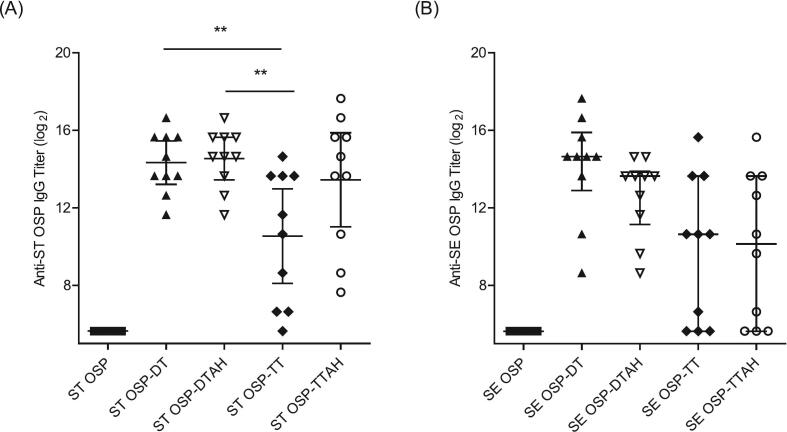
**Serum *S.* Typhimurium or *S.* Enteritidis OSP-specific IgG induced by various formulations with different carrier proteins and conjugation methods.** CD1 female mice (*n* = 10 per group) were immunized with 5 μg of OSP from either (A) ST OSP, ST OSP-DT, ST OSP-DTAH, ST OSP-TT, or ST OSP-TTAH, and (B) SE OSP, SE-OSP DT, SE OSP-DTAH, SE OSP-TT, or SE OSP-TTAH on days 0, 14, and 28 intramuscularly. Anti-ST OSP or -SE OSP IgG was assessed by ELISA in serum samples on day 56. The error bars represent the 95 % confidence intervals of geometric means or medians with the interquartile range. Multigroup comparison was performed using GraphPad Prism 10 software by either one-way ANOVA with Tukey's post-hoc test or Kruskal–Wallis analysis with Dunn's post-hoc test. * *P* < 0.05, ** *P* < 0.01, *** *P* < 0.001.

Eight batches of ST and SE OSPs conjugated to DT, DTAH, TT, or TTAH were formulated in PBS and evaluated for immunogenicity. ELISA results showed that the SE and ST OSP conjugates with DT induced 100 % seroconversion in immunized mice (data not shown), and the OSP conjugates with DT (ST OSP-DT and SE OSP-DT) generated significantly higher anti-OSP IgG titers than those conjugated to TT, indicating a superior immune response with DT as the carrier protein (Fig. 1). Interestingly, the presence of a hydrazide linker (–AH) did not consistently enhance immunogenicity. Both ST and SE OSP-DTs (without the linker) induced equal or higher anti-OSP IgG titers than their respective conjugates with the linker (DTAH) Table 1, Fig. 1.

**Table 1:** Characterization of iNTS OSP Conjugates with DT and TT.

Conjugation Lot	Mol. Size (Avg. kDa)	Final OSP:Pr ratio (w/w) in conjugate	Yield (%)	
			OSP	Protein
***Salmonella* Typhimurium OSP conjugate**				
ST OSP–DT	1498	1.11	64	58
ST OSP–DTAH	1755	1.07	81	76
ST OSP–TT	2282	0.78	60	76
ST OSP–TTAH	4560	0.79	66	84
***Salmonella* Enteritidis OSP conjugate**				
SE OSP–DT	1373	1.09	79	72
SE OSP–DTAH	2160	1.15	90	78
SE OSP–TT	2550	0.92	70	76
SE OSP–TTAH	5871	0.97	73	75

### 3.2 Determination of optimal conjugation reaction ratios for iNTS OSP conjugates

Based on the results of the carrier protein selection test for iNTS OSP conjugates, both SE and ST OSP conjugates were developed using DT as the carrier protein without linker modifications. The conjugates were prepared under two different CDAP reaction conditions with three different DT-to-OSP weight ratios of 0.5, 1, and 1.5. The details of the preparation and characterization of each iNTS ST and SE OSP conjugate are presented in Table 2. The results showed distinct molecular weight distributions for ST and SE OSP conjugates, despite the comparable sizes of ST and SE OSP, which averaged approximately 31 kDa for ST OSP and 28 kDa for SE OSP, as determined by SEC-MALS. ST OSP conjugates had a molecular weight range of 408–878 kDa, whereas SE OSP conjugates exhibited a broader range of 725–2786 kDa. This difference suggests that SE OSP conjugates form larger molecular complexes than ST OSP conjugates do under the same conjugation conditions.

An increase in the DT-to-OSP ratio led to a corresponding increase in the molecular weight of the conjugates. Similarly, higher CDAP input ratios resulted in higher molecular weights. This trend was consistent for both ST and SE OSP conjugates, indicating that the amount of carrier protein and the concentration of CDAP were critical factors influencing the final size of the conjugates. Regarding the yield of the conjugation process, the data showed that increasing the DT input ratio resulted in a more significant increase in the OSP yield than an increase in the CDAP input ratio. The highest yields were observed for the ST OSP-DT 2–3 and SE OSP-DT 2–3 batches, with yields of 79.3 % and 64.8 %, respectively. These findings demonstrate the importance of optimizing both the carrier protein and CDAP ratios to maximize conjugate production.

**Fig. 2:**
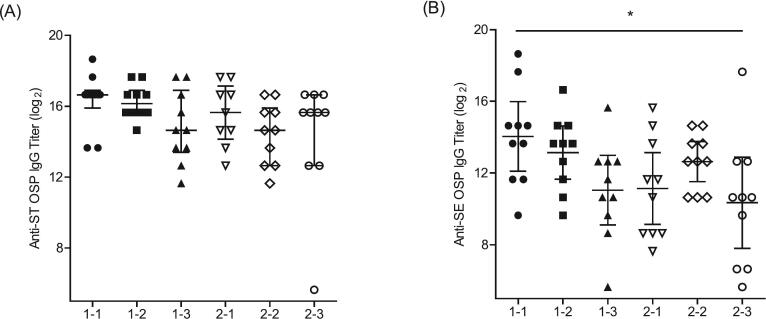
**Serum *S.*** T**yphimurium or *S.*** E**nteritidis OSP-specific IgG induced by various formulation with different reaction ratios of OSP: CDAP: DT carrier protein.** CD1 female mice (*n* = 10 per group) were immunized with 5 μg of OSP from either (A) ST OSP-DT conjugates or (B) SE OSP-DT conjugates on days 0, 14, and 28 intramuscularly. Anti-ST OSP or -SE OSP IgG was assessed by ELISA in serum samples on day 56. The error bars represent 95 % confidence intervals of geometric means or medians with the interquartile range. Multigroup comparison was performed using GraphPad Prism 10 software by either one-way ANOVA with Tukey's post-hoc test or Kruskal-Wallis analysis with Dunn's post-hoc test. * *P* < 0.05, ** *P* < 0.01, *** *P* < 0.001.

Six batches of each iNTS ST and SE OSP conjugate covering a range of molecular weights were evaluated for immunogenicity in mice (Fig. 2). Both the ST and SE OSP conjugates showed an inverse correlation between the molecular weight of the conjugate and immunogenicity. Specifically, a low molecular weight batch of ST OSP-DT 1–1 (avg. 408 kDa) induced the highest anti-ST OSP titers that were 4-fold higher than those of the high molecular weight batch ST OSP-DT 2–3 (avg. 878 kDa). Similarly, the anti-SE OSP titers were approximately 13-fold higher in the SE OSP-DT 1–1 batch (avg. 725 kDa) than in the SE OSP-DT 2–3 batch (avg. 2786 kDa). These results indicated that a lower molecular weight, associated with a lower ratio of DT protein input during conjugation, enhanced immunogenicity. The immunogenicity data further suggested that an OSP:CDAP:DT ratio of 1:1:0.5, as observed in the ST OSP-DT 1–1 and SE OSP-DT 1–1 batches, was optimal for inducing strong immune responses in both the ST and SE OSP conjugates. Although the ST OSP-DT 1–2 batch, prepared with a 1:1:1 OSP:CDAP:DT ratio, did not show a significant difference in immunogenicity compared to the 1:1:0.5 ratio (ST OSP-DT 1–1), its higher OSP yield (55.9 %) suggests that it may also be the yield, along with immunogenicity, that would need to be considered while selecting the conjugates in vaccines, underscoring the importance of optimizing both molecular size and the ratio of components in the development of conjugate vaccines Table 2, Fig. 2.

**Table 2:** Characterization of iNTS OSP conjugate batches with various CDAP and DT ratios.

iNTS OSP Conjugate Batch		Reaction ratio to OSP (w:w)		Yield (%) of conjugation		OSP:DT ratio in final product (w/w)	Mol. size (Avg. kDa)
		CDAP	DT	OSP	DT		
ST	ST OSP-DT 1–1	1:1	1:0.5	41.1	68.4	1.20	408
	ST OSP-DT 1–2	1:1	1:1	55.9	69.9	0.80	590
	ST OSP-DT 1–3	1:1	1:1.5	62.4	61.5	0.68	673
	ST OSP-DT 2–1	1:1.5	1:0.5	46.0	79.3	1.16	471
	ST OSP-DT 2–2	1:1.5	1:1	61.2	71.9	0.85	797
	ST OSP-DT 2–3	1:1.5	1:1.5	64.8	65.4	0.66	878
SE	SE OSP-DT 1–1	1:1	1:0.5	51.7	69.7	1.49	725
	SE OSP-DT 1–2	1:1	1:1	69.1	72.8	0.95	1021
	SE OSP-DT 1–3	1:1	1:1.5	74.2	68.3	0.72	1714
	SE OSP-DT 2–1	1:1.5	1:0.5	53.6	73.2	1.47	903
	SE OSP-DT 2–2	1:1.5	1:1	68.0	70.6	0.96	2256
	SE OSP-DT 2–3	1:1.5	1:1.5	79.3	74.5	0.71	2786

### 3.3 iNTS bivalent and trivalent formulation

To determine the optimal formulations of bivalent iNTS and trivalent iNTS/typhoid vaccines, various bivalent and trivalent formulations, including the Vi-DT typhoid conjugate vaccine, were evaluated. Optimal batches of the ST OSP conjugate (ST OSP-DT 1–2) and SE OSP-DT conjugate (SE OSP-DT 1–1) were selected for dose optimization in the monovalent formulation. Based on the optimal doses identified for both monovalent iNTS OSP conjugates, bivalent and trivalent conjugates were formulated to assess their immunogenicity (Table 3).

**Fig. 3:**
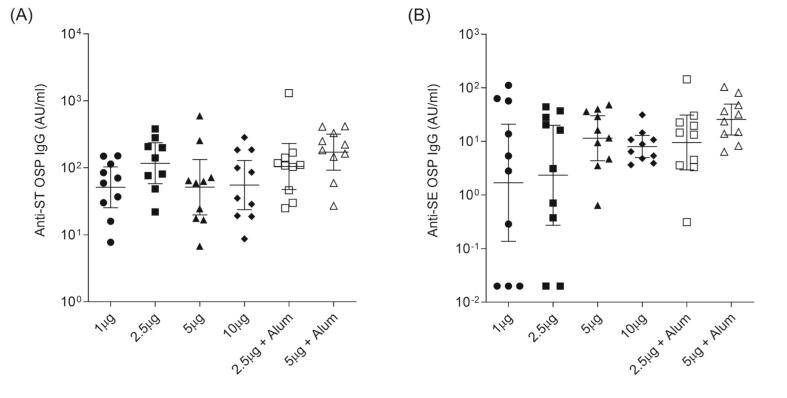
**Serum *S.* Typhimurium or *S.*** E**nteritidis OSP-specific IgG induced by different vaccine doses (1, 2.5, 5** and **10** μ**g) and alum adjuvanted formulation.** CD1 female mice (*n* = 10 per group) were immunized with (A) ST OSP-DT conjugates and (B) SE OSP-DT conjugates on days 0, 14, and 28 intramuscularly. Anti-ST OSP or -SE OSP IgG was assessed by ELISA in serum samples on day 56. The error bars represent the 95 % confidence intervals of geometric means. Multigroup comparison was performed using GraphPad Prism 10 software by one-way ANOVA with Tukey's post-hoc test. * *P* < 0.05, ** *P* < 0.01, *** *P* < 0.001.

Monovalent formulations of each ST and SE OSP conjugates were tested at doses of 1, 2.5, 5, and 10 μg without adjuvants and at 2.5 and 5 μg with alum phosphate adjuvant. The results demonstrated that ST OSP and SE OSP conjugates induced high levels of immunogenicity, particularly at doses of 2.5 and 5 μg, respectively (Fig. 3). Addition of the alum adjuvant further enhanced the immune response, especially against the SE OSP conjugates (Fig. 3B).

**Fig. 4:**
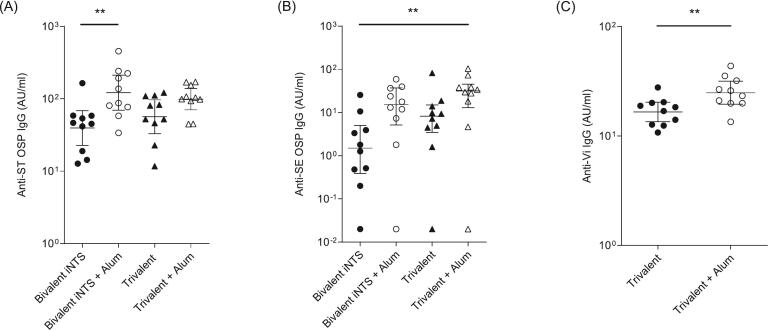
**Serum *S.*** T**yphimurium, *S.* Enteritidis OSP-, or *S.* Typhi Vi-specific IgG induced by bivalent iNTS and trivalent (iNTS/Typhoid) vaccine candidates in the absence or presence of alum.** CD1 female mice (n = 10 per group) were immunized with bivalent iNTS including 2.5 μg of ST OSP-DT and 5 μg of SE OSP-DT or trivalent formulation including bivalent iNTS plus 2.5 μg of Vi-DT on days 0, 14 and 28 intramuscularly. Anti-ST OSP, -SE OSP, or -Vi IgG were assessed by ELISA in serum samples on day 56. The error bars represent 95 % confidence intervals of the geometric means. Multigroup comparison was performed using GraphPad Prism 10 software by either one-way ANOVA with Tukey's post-hoc test or Kruskal-Wallis analysis with Dunn's post-hoc test for anti-ST and -SE OSP IgG. A two-tailed *t*-test was used to compare anti-Vi IgG levels. * *P* < 0.05, ** *P* < 0.01, *** *P* < 0.001.

Fig. 4 shows the anti-ST OSP IgG, anti-SE OSP IgG, and anti-Vi IgG titers, along with the seropositivity rates across the different vaccine formulations. The bivalent formulation with alum significantly increased anti-ST OSP IgG responses (Fig. 4A). Trivalent formulations, which included the Vi-DT conjugate in a multi-dose vial format with 2-phenoxyethanol as a preservative, elicited anti-ST OSP IgG responses comparable to those of the bivalent groups. Inclusion of the Vi-DT conjugate did not negatively affect the immunogenicity of the ST OSP-DT conjugates. For anti-SE OSP IgG titers, the alum-adjuvanted bivalent formulation induced a higher response than the non-adjuvanted formulation. In the trivalent formulations, both alum-adjuvanted groups exhibited higher anti-SE IgG titers than the bivalent formulations with alum and non-adjuvanted trivalent conjugates (Fig. 4B).

Anti-ST and -SE OSP IgG responses were significantly lower for the bivalent and trivalent formulations than for each monovalent conjugate, particularly for the bivalent conjugates, as shown in Figs. 3, 4 and Table 4, respectively. The geometric mean titer (GMTs) of anti-ST OSP IgG was 117. 1 AU/mL for the ST monovalent conjugate at 2.5 μg, 39.4 A U/mL for the bivalent formulation, and 56.5 AU/mL for the trivalent conjugates. For anti-SE OSP IgG, the GMTs were 11.5 AU/mL for the SE monovalent conjugate at 5 μg, 1.2 AU/mL for the bivalent formulation, and 5.0 AU/mL for the trivalent formulations. However, the addition of alum phosphate adjuvant in the bivalent and trivalent formulations increased the anti-ST OSP IgG levels to 121.3 and 99.1 AU/mL, respectively, and the anti-SE OSP IgG levels to 8.3 and 14.9 AU/mL, respectively.

The trivalent formulation with alum also elicited higher anti-Vi IgG titers than the non-alum formulation (Fig. 4C), demonstrating the benefit of including an adjuvant to enhance the immune response to the Vi antigen. The seropositivity rates were consistently high across anti-ST OSP, anti-SE OSP IgG, and anti-Vi IgG antibodies, indicating that both the bivalent and trivalent formulations were effective in inducing robust immune responses against their respective antigens Table 3, Table 4, Fig. 3, Fig. 4.

**Table 3:** Formulation test design of iNTS and typhoid conjugates.

Type	Test formulation	Adjuvant	Dose from conjugate
Monovalent	ST OSP-DT	w/o adjuvant	1, 2.5, 5, and 10 μg ST OSP
		Alum phosphate	2.5 and 5 μg ST OSP
	SE OSP-DT	w/o adjuvant	1, 2.5, 5, and 10 μg SE OSP
		Alum phosphate	2.5 and 5 μg SE OSP
Bivalent	ST OSP-DT + SE OSP-DT	w/o adjuvant	2.5 μg ST OSP
			5 μg SE OSP
		Alum phosphate	2.5 μg ST OSP
			5 μg SE OSP
Trivalent⁎	ST OSP-DT + SE OSP-DT + Vi-DT	w/o adjuvant	2.5 μg ST OSP
			5 μg SE OSP
			2.5 μg Vi
		Alum phosphate	2.5 μg ST OSP
			5 μg SE OSP
			2.5 μg Vi

⁎ Multi-dose vial formulation with 2-Phenoxyethanol as preservative.

**Table 4:** Geometric mean titer (GMT) of anti-ST OSP IgG,-SE OSP IgG, and -Vi IgG in monovalent, bivalent, and trivalent formulations.

Type	Monovalent	Bivalent	Trivalent	Bivalent + alum	Trivalent + alum
	GMT⁎ (95 % CI)				
ST OSP IgG	117.1 (58.0, 236.5)	39.4 (22.7, 68.4)	56.5 (33.0, 96.8)	121.3 (69.4, 212.0)	99.1 (70.4, 139.4)
SE OSP IgG	11.5 (4.4, 30.2)	1.2 (0.3, 5.4)	5.0 (1.0, 24.2)	8.3 (1.6, 44.0)	14.9 (2.6, 87.0)
Vi IgG			16.6 (13.5, 20.4)		24.9 (19.5, 31.6)

⁎ Geometric Mean Titers (unit: AU/ml); CI: Confidence interval.

## 4 Discussion

Several factors influence the immunogenicity of glycoconjugate vaccines, including carrier protein, conjugation method, chemical modification by linkers, the loading amount or ratio of polysaccharide to the carrier protein, and the physical properties, such as the molecular weight of the glycoconjugate achieved through cross-linking. [[23], [24], [25], [26]]. In this study, we aimed to optimize the conjugation process by systematically evaluating different carrier proteins, DT and TT. Both are cost-effective and easy to process, and capable of achieving high conjugation yield, making them commonly used in affordable conjugate vaccines for LMICs. Our findings identified DT as the carrier protein that induces significantly higher anti-OSP immunogenicity compared to TT in both iNTS SE and SE OSP conjugates. Consequently, we focused on optimizing the CDAP conjugation process for iNTS OSP conjugation using DT as carrier protein. All monovalent iNTS conjugates generated significantly higher anti-ST and anti-SE OSP IgG responses than the unconjugated OSP antigens in all mice. Notably, all mice achieved complete seroconversion (≥4-fold increase compared to baseline, data not shown) when DT was used as the carrier protein, and induced higher anti-OSP IgG responses in both ST & SE-OSP conjugates compared to TT. In another glycoconjugate vaccine study, similar results were observed, with a significant increase in anti-polysaccharide IgG and rabbit serum bactericidal antibody (SBA) responses to serogroup A meningococcal (Men A) saccharide-DT conjugates and MenA-CRM_197_ compared to MenA-TT [27]. However, in a clinical study of typhoid conjugate vaccines, comparable immunogenicity between Vi-DT and Vi-TT conjugate vaccines was observed in a Nepalese phase 3 trial in terms of the seroconversion rate and GMT of anti-Vi IgG [28].

In the optimization batch tests of the CDAP-to-polysaccharide and carrier protein-to-polysaccharide ratios, our findings showed that a lower molecular weight, achieved using a lower CDAP-to-polysaccharide and carrier protein-to-polysaccharide ratio, induced higher immunogenicity compared to the larger molecular weight of OSP conjugates produced with higher CDAP and protein-to-carbohydrate ratios. These observations may be attributed to epitope masking or blocking of attachment sites on the OSP caused by excessive crosslinking of the linkers on the OSP conjugate [29,30]. Similar results were observed in an iNTS conjugation study in which Stefanetti et al. reported that ST conjugates prepared using a random conjugation method via periodate oxidation showed decreased efficacy as the degree of ST carbohydrate chain derivatization increased [31]. This is in contrast to the typhoid Vi polysaccharide-conjugate vaccine, which involves a relatively large polysaccharide. In our previous study [32], the immunogenicity of the Vi polysaccharide in the conjugate increased as the size of the conjugate increased owing to cross-linking. However, for smaller polysaccharides such as iNTS OSPs, the immunogenicity decreased with increased cross-linking, likely due to reduced epitope exposure.

We also examined the effects of the carrier protein modification. These results suggest that while the inclusion of a hydrazide linker increases the molecular weight of the conjugates, it does not necessarily enhance immunogenicity. From a process perspective, this direct conjugation method without carrier protein modification offers a cost-effective approach for producing iNTS conjugate vaccines. This eliminates the need for additional modification and post-modification purification steps, while achieving sufficiently high OSP conjugation yields of over 50 % when DT is conjugated to OSPs at the optimal reaction ratio. Compared to other vaccine candidates, such as iNTS OSP-FliC conjugates and GMMA-based iNTS vaccine candidates [16,17,21,33], the approach in this study offers distinct advantages in terms of vaccine technology accessibility and availability in LMICs, the primary regions where the disease is endemic. This is due to the processability and use of chemical conjugation with existing licensed conjugate vaccines and carrier proteins. Based on this process optimization study, we successfully produced consistent iNTS conjugate batches at gram-scale, demonstrating the scalability of CDAP conjugation chemistry for large-scale iNTS conjugate vaccine production. Additionally, the technology transfer to a vaccine manufacturer was successfully completed, enabling the production of toxicology study batches under Good Laboratory Practice (GLP) standards. The transferred iNTS conjugate and trivalent vaccine manufacturing processes demonstrated batch consistency, with the stability of the monovalent and bivalent drug substances, as well as the trivalent vaccine drug product, maintained for 24 months under long-term storage at 5 ± 3 °C (data not shown).

The anti-ST and anti-SE OSP IgG responses of each monovalent candidate were assessed to determine the optimal formulation of the bivalent iNTS vaccine. Additionally, the bivalent formulation was combined with a typhoid Vi-DT conjugate vaccine to evaluate its feasibility as a trivalent formulation. Antibody responses peaked at 2.5 μg for ST OSP-DT and 5 μg of SE OSP-DT conjugates, identifying these as the optimal dose for the monovalent vaccines. The addition of aluminum phosphate to both bivalent and trivalent formulations significantly enhanced anti-ST OSP, anti-SE OSP IgG, and anti-Vi IgG responses, including the trivalent vaccine that incorporated the typhoid Vi-DT conjugate vaccine. We found that anti-ST OSP IgG and anti-SE OSP IgG responses significantly inhibited each other in the bivalent and trivalent formulations compared with each monovalent conjugate. However, immune interference in the bivalent formulation was eliminated by the addition of aluminum phosphate, which restored the antibody titers to the levels observed with each monovalent conjugate. Additionally, the inclusion of alum in the trivalent formulation restored the anti-Vi response to a level comparable to the monovalent Vi-DT conjugate. Aluminum salts influence the immunogenicity and stability of glycoconjugate vaccines by interacting with the protein moiety of glycoconjugate [34]. Although aluminum salts are the only adjuvants used in licensed glycoconjugate vaccines such as those for *Haemophilus influenzae* type b (Hib) and multivalent pneumococcal conjugate vaccines (PCV) [35], evidence supporting the elimination of interference by the addition of alum in multivalent vaccines is limited. Immune interference of carbohydrate-specific antibody responses in multivalent PCVs was observed when CRM_197_ was used as the carrier protein, with the extent of interference varying depending on the serotype. This may be due to multiple polysaccharide-specific B cells competing for T helper cells owing to the use of the same carrier proteins, which can be resolved by adjusting the carbohydrate-to-protein ratio [36]. Contrary to our findings, Fiorino et al. reported no interference in IgG responses between *S.* Typhimurium and *S.* Enteritidis in the iNTS OSP-CRM_197_ bivalent formulation, although alum enhanced antibody responses to bivalent vaccines compared to the unadjuvanted formulation [37]. The discrepancy between these two studies could be due to various factors, including differences in vaccine formulation, specific strains used for OSP purification, carrier proteins, and conjugation methods.

One limitation of the study is the focus on immunogenicity rather than direct protective efficacy, which requires further investigation in clinical trials. Additionally, while the murine model provides valuable insights, human immune responses can differ significantly, necessitating careful interpretation of these results when extrapolating to potential human applications. Functional antibodies such as serum bactericidal and opsonophagocytic antibodies, have been used as correlation of protection against bacterial diseases including Hib [38], Meningococcal [39], and pneumococcal diseases [40]. However, functional antibodies against *Salmonella* species may not serve as surrogate markers et for protection against bacterial infection. Higginson et al, found that a live-attenuated *S.* Typhimurium vaccine candidate (CVD 1926) induced opsonophagocytic antibody and showed 80 % of vaccine efficacy in a gastroenteritis model of rhesus macaque [41]. Moderate diarrheal disease was observed in one out of five animals, despite this animal generating the highest functional antibody titer [41]. Additionally, serum bactericidal antibodies were not able to provide protection against typhoid fever in the controlled human infection model, although disease severity was reduced by functional antibodies [42].

The multivalent combination strategy mirrors the success of multivalent pneumococcal conjugate vaccines, which effectively reduced pneumococcal infections by combining the polysaccharide conjugates for multiple serotypes [43,44]. Further with this concept, this trivalent vaccine includes a typhoid conjugate vaccine into a single formulation, offering additional preventive effects and expanding its utility against multiple diseases, thereby increasing its demand and supporting its cost-effectiveness by streaming production processes and reducing overall manufacturing cost compared to monovalent vaccine [10]. Additionally, this strategy can simplify logistics and distribution for the vaccination to reduce healthcare burden of *Salmonella* infections, especially in resource-limited settings. These advantages aligned with frameworks such as the “Full Value Vaccine Assessment” (FVVA) for iNTS disease [45], which helps define the value position of an iNTS vaccine and guide its development strategy. FVVA emphasizes that the trivalent vaccine has the potential to surpass the stand-alone iNTS vaccine, which faces challenges due to potentially limited market demand. Furthermore, the trivalent vaccine serves as a practical tool to overcome hurdles such as regulatory approval processes, field implementation strategies, and commercial viability of the iNTS vaccine.

In conclusion, our study identified key process parameters to develop the iNTS conjugate vaccine through process optimization for high-efficiency and low-cost production. The optimized iNTS conjugate vaccine, combined with its trivalent formulation alongside the typhoid conjugate vaccine, demonstrated robust immunogenicity for each of the three antigens in preclinical studies. This approach offers a promising solution for protection against iNTS and typhoid fever, addressing a critical public health need in endemic areas with limited resources.

## CRediT authorship contribution statement

**So Jung An:** Writing – review & editing, Writing – original draft, Investigation, Formal analysis. **Jae Seung Yang:** Writing – review & editing, Writing – original draft, Investigation, Formal analysis. **Myung Hwa Chae:** Formal analysis. **Joo Sung Woo:** Formal analysis. **Ye Eun Kang:** Formal analysis. **Ravi Ganapathy:** Writing – review & editing, Supervision. **Ruchir Kumar Pansuriya:** Writing – review & editing, Resources. **Jung Ah Choi:** Writing – review & editing, Formal analysis. **Yeon Kyung Yoon:** Formal analysis. **Eugene Lee:** Formal analysis. **Seul Bee Lee:** Formal analysis. **Gaurav Pandey:** Writing – review & editing, Supervision. **Ji Won Lee:** Formal analysis. **Ji Soo Lee:** Formal analysis. **So Hee Bae:** Formal analysis. **Soh-Won Kweon:** Formal analysis. **Soo Ji Kim:** Formal analysis. **Seung Han Seon:** Formal analysis. **Jerome H. Kim:** Supervision, Conceptualization. **Manki Song:** Writing – review & editing, Supervision.

## Declaration of competing interest

The authors declare that they have no known competing financial interests or personal relationships that could have appeared to influence the work reported in this paper.

## Data Availability

Data will be made available on request.

## Appendix A Supplementary data

Supplementary material: **Fig. S1. Carrier protein modification.** The carrier protein (DT or TT) was modified at its carboxylic acid group using adipic acid dihydrazide (ADH). This modification was achieved by reacting the carrier protein with 1-ethyl-3-(3-dimethylaminopropyl) carbodiimide (EDC) in the presence of ADH, resulting in the formation of DTAH and TTAH. **Fig. S2. Synthetic scheme for iNTS OSP conjugation by method 1 and method 2.** (A) Activation of hydroxyl groups on OSP for both OSP conjugation methods 1 and 2, (B) In method 1, the activated OSP was coupled to the ADH modified carrier protein (DTAH or TTAH), and (C) In method 2, the activated OSP was coupled to the unmodified carrier protein (DT or TT). Image 1
