# Supplementary material for: Development of invasive non-typhoidal Salmonella conjugate vaccines and their evaluation in a trivalent formulation with typhoid conjugate vaccine
Source: Vaccine. 2025 Apr 11;52:None. doi: 10.1016/j.vaccine.2025.126913 (PMC11997612; doi:10.1016/j.vaccine.2025.126913)
Supplement: Supplementary file 1 — Supplementary material: Fig. S1. Carrier protein modification. The carrier protein (DT or TT) was modified at its carboxylic acid group using adipic acid dihydrazide (ADH). This modification was achieved by reacting the carrier protein with 1-ethyl-3-(3-dimethylaminopropyl) carbodiimide (EDC) in the presence of ADH, resulting in the formation of DTAH and TTAH. Fig. S2. Synthetic scheme for iNTS OSP conjugation by method 1 and method 2. (A) Activation of hydroxyl groups on OSP for both OSP conjugation methods 1 and 2, (B) In method 1, the activated OSP was coupled to the ADH modified carrier protein (DTAH or TTAH), and (C) In method 2, the activated OSP was coupled to the unmodified carrier protein (DT or TT). [file mmc1.docx]

**Supplementary material**

**Title:**

**Development of Invasive Non-typhoidal Salmonella Conjugate Vaccines and Their Evaluation in a Trivalent Formulation with Typhoid Conjugate Vaccine**

**Running title:**

**iNTS conjugate vaccine, Trivalent iNTS/Typhoid Conjugate Vaccine**

**Authors:**

So Jung An^a#^, Jae Seung Yang^a#^, Myung Hwa Chae^a^, Joo Sung Woo^a^, Ye Eun Kang^a^, Ravi Ganapathy^b^, Ruchir Kumar Pansuriya^a^, Jung Ah Choi^a^, Yeon Kyung Yoon^a^, Eugene Lee^a^, Seul Bee Lee^a^, Gaurav Pandey^a^, Ji Won Lee^a^, Ji Soo Lee^a^, So Hee Bae^a^, Soh-Won Kweon^a^, Soo Ji Kim^a^, Seung Han Seon^a^, Jerome H. Kim^a^, Manki Song^a*^

^a^ International Vaccine Institute, SNU Research Park, 1 Gwanak-ro, Gwanak-gu, Seoul, Republic of Korea

^b^ Hilleman Laboratories Singapore Pte Ltd, 21 Biopolis Rd, #04-06/12 Nucleos North Tower, Singapore

^#^ contributed equally as first authors

**Synthesis and characterization of iNTS OSP Conjugates**

The chemical conjugation of *S.* Typhimurium and *S.* Enteritidis OSP to carrier proteins was carried out using an organic cyanylating reagent, CDAP, to activate the OSP for coupling with the carrier protein, DT or TT. This process was using two different CDAP conjugation strategies, either with or without carrier protein modification (Fig. S1 and S2). In Method 1 (indirect conjugation), DT or TT was used as the carrier protein and modified at its carboxylic acid group with adipic acid dihydrazide (ADH, Sigma Aldrich). This modification was achieved by reacting the carrier protein with 1-ethyl-3-(3-dimethylaminopropyl) carbodiimide (EDC, Sigma Aldrich) in the presence of ADH, following a modified version of the method described by Kossaczka *et al*. [1] (Fig. S1). The resulting ADH-modified carrier proteins, referred to as DTAH or TTAH, were subsequently coupled to OSP in presence of CDAP (Fig. S2, A and B) [2-3]. In Method 2 (direct conjugation without protein modification), DT or TT, used as carrier protein, was directly coupled to OSP modified without linker modification (Fig. S2, A and C). Both methods resulted in iNTS OSP conjugates.

**Figure S1: Carrier protein modification**

| 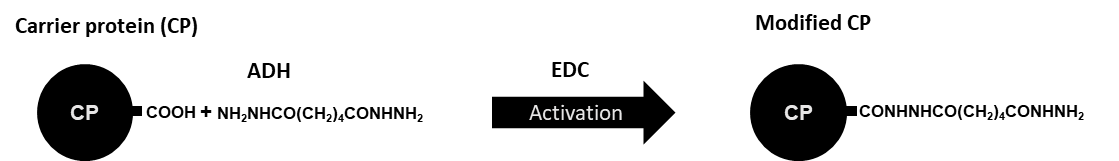 |
| --- |

**Figure S2: Synthetic scheme for iNTS OSP conjugation by method 1 and method 2**

**Synthesis of iNTS OSP conjugates by method 1 and method 2:**

*Method 1: Conjugation of OSP to ADH-modified carrier protein (indirect)*

Two ST OSP conjugates and two SE OSP conjugates batches were generated using DTAH and TTAH through indirect CDAP conjugation. For ADH modification, the carrier protein (DT or TT) was prepared in 100 mM 2-(*N*-morpholino) ethanesulfonic acid (MES) buffer at pH 5.6. For DTAH, 0.16 mM of DT (50 mg/mL) or for TTAH, 0.067 mM of TT (50 mg/mL) was prepared. ADH (90 mg/mL in MES buffer) was slowly added to the solution and stirred for 3 minutes, followed by the addition of EDC (20 mg/mL in MES buffer) to the reaction mixture. The reaction was performed with final concentrations of 10 mg/ml carrier protein, 200 mM ADH and either 10 mM or 20 mM EDC. The final weight ratios of carrier protein, EDC, and ADH were 10:2:35 for DT modification and 10:4:35 for TT modification. After 1 hour stirring at room temperature, the reaction mixture was dialyzed using 6-8 kDa MWCO dialysis tube against PBS, pH 7.4 at 4º C with 3 changes of buffer at 3-hour intervals. After dialysis, the protein concentration was measured using the Lowry assay and found to range from 7.5 to 8.5 mg/mL. The ADH concentration of the modified carrier protein was determined using a colorimetric TNBS assay [4]. DTAH and TTAH had their carboxylic acid groups modified with ADH, achieving conversion rates of 4.0 % and 3.9 % respectively relative to the total protein amount.

SE or ST OSP was prepared at a concentration of 10 mg/mL in 10 mM sodium borate buffer and CDAP was prepared at a concentration of 100 mg/mL (425.6 mM) in acetonitrile. CDAP was added to OSP at 1:1 weight ratio for each DTAH and TTAH conjugation batch with SE and ST OSP (refer to Table-1 in the manuscript) or at 1:1 and 1:1.5 weight ratios for various CDAP ratio batches (refer to Table-2 in the manuscript). The mixture was stirred for 30–40 seconds, after which 0.5 N sodium hydroxide was added to adjust the pH to 9.5 ± 0.1, and this pH was maintained for 2 minutes. Next, the ADH-modified carrier protein was added to the OSP at a 1:1 weight ratio for the carrier protein selection batches (Table-1 in the manuscript). The reaction mixture was stirred for 2 hours at room temperature. Following the conjugation reaction, any unreacted cyanate ester groups were quenched by adding an equal volume of 2 M glycine (pH 8.0) to the reaction mixture and stirring for an additional 30 minutes. The purification process to remove unconjugated OSP and carrier proteins was performed using a TFF System (KrosFlo® KR2i, Repligen, MA, USA) in PBS (pH 7.4). OSP-DT conjugates were purified using the 100-kDa MWCO Spectrum® mPES hollow fiber filter module, whereas OSP-TT conjugates were purified using a 300-kDa MWCO mPES hollow fiber filter. All conjugates were sterile filtered using a 0.2 µm filter (Sartopore 2, Sartorius, Göttingen, Germany) before formulations.

*Method 2: Conjugation of OSP to unmodified carrier protein (direct)*

Four different conjugates were generated using unmodified DT or TT (referred to DT and TT) for carrier protein selection batches (Table-1 in the manuscript), and six batches each of ST and SE OSP-DT conjugates were prepared to evaluate various CDAP and DT ratios (Table-2 in the manuscript).

The carrier protein was prepared in PBS (pH 7.4) to greater than 10 mg/ml using 30 kDa (MWCO) TFF filtration. ST or SE OSP was prepared at a concentration of 10 mg/mL in 10 mM sodium borate buffer, while CDAP was prepared at 100 mg/mL (425.6 mM) in acetonitrile. For the carrier protein selection batches, CDAP was added to OSP at a 1:1 weight ratio. For the various process optimization ratio batches (Table-2 in the manuscript), CDAP was added to the OSP at an OSP: CDAP weight ratio of either 1:1 or 1:1.5. After stirring the mixture for 30–40 seconds, 0.5 N sodium hydroxide was added to adjust the pH to 9.5 ± 0.1, and this pH was maintained for 2 minutes. For all carrier protein selection batches, DT or TT was added at a 1:1 weight ratio of OSP in the mixture. For the various process optimization ratio batches, OSP:protein weight ratios of 1:0.5, 1:1 or 1:1.5 were used, followed by stirring for 2 hours at room temperature. After the reaction, any unreacted cyanate ester groups were quenched by adding an equal volume of 2 M glycine (pH 8.0) to the reaction mixture and stirring for an additional 30 minutes.

The purification process to remove unconjugated OSP and carrier proteins was performed using a TFF System (KrosFlo® KR2i, Repligen, MA, USA) in PBS (pH 7.4). OSP-DT conjugates were purified using the 100-kDa MWCO Spectrum® mPES hollow fiber filter module, whereas OSP-TT conjugates were purified using a 300-kDa MWCO mPES hollow fiber filter. All conjugates were sterile filtered using a 0.2 µm filter (Sartopore 2, Sartorius, Göttingen, Germany) before formulations.

References:

1. Kossaczka Z, Bystricky S, Bryla,DA, Shiloach J, Robbins JB, Szu SC. Synthesis and immunological properties of Vi and di-O-acetyl pectin protein conjugates with adipic acid dihydrazide as the linker. Infect Immun 1997;65:2088-93.

2. Simon, R., et al., Salmonella enterica serovar enteritidis core O polysaccharide conjugated to H:g,m flagellin as a candidate vaccine for protection against invasive infection with S. enteritidis. Infect Immun, 2011. 79(10): p. 4240-9.

3. Baliban, S.M., et al., Development of a glycoconjugate vaccine to prevent invasive Salmonella Typhimurium infections in sub-Saharan Africa. PLOS Neglected Tropical Diseases, 2017. 11(4): p. e0005493.

4. Szu SC, Stone AL, Robbins JD, Schneerson R, Robbins JB. Vi capsular polysaccharide-protein conjugates for prevention of typhoid fever. Preparation, characterization, and immunogenicity in laboratory animals. J Exp Med 1987;166:1510-24.
